# Cost‐of‐illness of metastatic gastroenteropancreatic neuroendocrine tumours in Sweden—A population‐based register‐linkage study

**DOI:** 10.1111/ecc.12983

**Published:** 2019-01-16

**Authors:** Eva Lesén, Daniel Granfeldt, Aude Houchard, Anthony Berthon, Jérôme Dinet, Sylvie Gabriel, Åse Björstad, Ingela Björholt, Anna‐Karin Elf, Viktor Johanson

**Affiliations:** ^1^ Nordic Health Economics Gothenburg Sweden; ^2^ PharmaLex Sweden, formerly Nordic Health Economics Gothenburg Sweden; ^3^ Ipsen Boulogne‐Billancourt France; ^4^ Department of Surgery Sahlgrenska University Hospital Gothenburg Sweden

**Keywords:** cost‐of‐illness, GEP‐NET, metastatic, register study, Sweden, treatment

## Abstract

The objective was to estimate the cost‐of‐illness of grades 1 and 2 metastatic gastroenteropancreatic neuroendocrine tumours (GEP‐NETs) in Sweden in 2013 in a population‐based study including all patients diagnosed between 2005 and 2013. Data were obtained from national registers, and patients who utilised healthcare resources due to metastatic GEP‐NETs in 2013 were included. The study included 478 patients (mean age 64 [*SD*=11] years, 51% men). The majority (80%) had small intestinal NET, 10% had pancreatic NET, and 41% had carcinoid syndrome. The total cost‐of‐illness was €12,189,000 in 2013, of which direct costs constituted 77% and costs from production loss constituted 22%. The largest contributor to the direct medical costs was prescription drugs (54%; primarily somatostatin analogues [91% of the total drug cost]). Production loss due to sickness absence constituted 52% of the total costs of production loss. The total annual cost per patient was €25,500. By patient group, the cost was €24,800 (95% CI €21,600–€28,100) for patients with small intestinal NET, €37,300 (95% CI €23,300–€51,300) for those with pancreatic NET and €18,600 (95% CI €12,600–€24,500) for patients with other GEP‐NETs. To conclude, the total annual cost of grades 1 and 2 metastatic GEP‐NETs in Sweden was €25,500 per patient and year.

## 1 INTRODUCTION

Gastroenteropancreatic neuroendocrine tumours (GEP‐NETs) are a heterogeneous group of neoplasms. These rare tumours are derived from the endocrine system in the gastrointestinal (GI) tract and pancreas and represent <2% of all GI tumours (Dasari et al., 2017; Lepage, Bouvier, & Faivre, 2013).

The clinical presentation of GEP‐NETs varies depending on, for example, tumour site and whether a hormonal hypersecretion is causing clinical symptoms, that is, whether the tumour is functioning or non‐functioning. In tumours of certain origins, for example, gastric and rectal, the tumours are most often indolent and very rarely metastatic (Frilling et al., 2014). Although the disease is often metastatic when diagnosed in other sites, such as the small intestine, it usually remains asymptomatic for many years. At clinical presentation, it can manifest with acute obstructive symptoms, for example, abdominal pain, nausea and vomiting, or symptoms caused by hormone hypersecretion or by metastases. Incorrect and delayed diagnosis is common (van Cutsem, 2013). The majority of patients (with the exception of patients with gastric and rectal NETs) have metastatic disease at the time of diagnosis (Niederle et al., 2016; Pavel et al., 2016). The median age at diagnosis is approximately 63 years, but it varies considerably depending on the tumour site (Ahmed et al., 2009; Lawrence et al., 2011; van Cutsem, 2013). The 5‐year survival rate is approximately 68% according to an American study, spanning from approximately 38% for pancreatic NETs to 89% for rectal NETs (Lawrence et al., 2011).

Surgery is the primary treatment for loco‐regional GEP‐NETs but should always also be considered in GEP‐NETs with distant metastases, usually hepatic metastases (Niederle et al., 2016). Other treatment options include the following: pharmacological treatment with somatostatin analogues (SSA); interferon alpha (IFN‐alpha); molecular targeted therapy (i.e., oncogenic pathway inhibitors) and chemotherapy; radiotherapy with peptide receptor radionuclide therapy (PRRT) or selective internal radiation therapy (SIRT); and ablative interventions, such as hepatic artery embolisation (HAE) and radiofrequency (RFA), or microwave ablation (Frilling et al., 2014; Massironi et al., 2008).

The objective was to estimate the cost‐of‐illness of metastatic GEP‐NETs (grade 1 or 2) in Sweden from a societal perspective.

## 2 METHODS

### 2.1 Data sources

Data were obtained via linkage of several national registers covering the entire Swedish population. Patients were selected via the Cancer Register, which includes information on all newly diagnosed tumours. The National Patient Register includes information on all specialised healthcare contacts, and this was used to complement information on metastatic status as well as healthcare resource use. The Swedish Prescribed Drug Register was used for data on prescribed medicines purchased in Swedish pharmacies. These registers are held by the National Board of Health and Welfare. Furthermore, the Register on Sickness Absence, managed by the Social Insurance Agency, was used for information on the time and underlying diagnosis for sickness absence periods lasting >14 days. The linkage between the registers was facilitated via the unique personal identification number.

### 2.2 Study population

Patients with a first diagnosis of metastatic GEP‐NET (grade 1 or 2) established in Sweden between 1 July 2005 and 31 December 2013 were included in the study. The start of the inclusion period was selected due to changes in the diagnostic coding system in the Cancer Register 2004/2005 and since the Drug Register was established in July 2005. The cost‐of‐illness assessment was performed for 2013; therefore, patients who died prior to 1 January 2013, as well as patients who did not utilise health care for metastatic GEP‐NETs, carcinoid syndrome or carcinoid heart disease in 2013, were not eligible for inclusion.

The following diagnostic criteria (both criteria were mandatory) were used to define GEP‐NETs according to data in the Cancer Register: (a) International Classification of Diseases for Oncology, 3rd edition (ICD‐O/3) codes for gastroenteropancreatic tumour sites: C16–C20 and C25; and (b) Systematised Nomenclature of Medicine, version 3 (SNOMED3) codes for neuroendocrine morphological type: 80133, 80413, 81500, 81503, 81510, 81513, 81521, 81523, 81531, 81533, 81553, 81561, 81563, 82403, 82413, 82421, 82423, 82463, 82493 and 86830. To account for potential variations in SNOMED3 coding concerning grade, codes suggestive of grade 3 disease were also included. Patients deemed to have grade 3 tumours based on survival analysis (described below) were excluded. Metastatic GEP‐NET disease at the time of diagnosis was identified based on tumour, node, metastases (TNM) codes in the Cancer Register (N1‐3 and/or M1) and/or the International Classification of Diseases, 10th revision (ICD‐10) codes in the National Patient Register (specialised healthcare visit/admission with diagnostic code C77–C79 within 6 months from the GEP‐NET diagnosis), and patients with metastatic GEP‐NET at the time of diagnosis were selected. The morphological codes registered at each tumour site were reviewed to remove patients with inconsistent or invalid diagnoses. Patients likely to have grade 3 tumours (morphological codes 80413 and 82463) were excluded on the basis of a comparison of their survival (via Kaplan–Meier curves and log‐rank tests) with the survival of patients with other morphological codes.

### 2.3 Cost‐of‐illness assessment

The cost‐of‐illness assessment encompassed the year 2013 and included direct medical costs (healthcare resource use and prescription drugs), direct non‐medical costs (transportation in connection with healthcare visits and admissions) and costs from production loss (absence during healthcare visits/admissions, sickness absence and mortality) due to metastatic GEP‐NET arising on or after the date of GEP‐NET diagnosis. Furthermore, healthcare resources and costs related to the diagnostic ascertainment of patients diagnosed in 2013 were included. All ICD‐10 codes; procedure codes for surgical, medical or diagnostic interventions; and Anatomical Therapeutic Chemical (ATC) codes for prescription drugs registered in 2013 were reviewed for inclusion.

Information on specialised healthcare use was obtained from the National Patient Register, and the costs were estimated based on the diagnosis‐related group (DRG) codes, which are registered for each healthcare visit/admission. The DRG code reflects the costs associated with the healthcare encounter and any interventions or procedures performed at that occasion. The weights for each DRG code were derived from the national weight lists for 2013 published by the National Board of Health and Welfare. The price for a DRG weight equal to 1 was €4,697 for 2015. To avoid the double counting of healthcare resource use when more than one intervention or procedure was performed at the same visit/admission, the following hierarchy was applied: (a) surgical intervention (i.e., if surgery had been performed, the healthcare visit/admission was categorised as surgery, irrespective of which other interventions had been performed at the same visit/admission), (b) medical interventions (HAE, RFA, PRRT, external radiotherapy and administration of pharmacological treatment), (c) diagnostic procedure (if performed prior to the date of diagnosis of patients diagnosed in 2013), (d) imaging and (e) examinations. The remaining healthcare visits and admissions that were related to metastatic GEP‐NET, carcinoid syndrome or carcinoid heart disease were classified as other outpatient visits/inpatient admissions, during which none of the above stated interventions or procedures had been performed.

The drugs related to GEP‐NETs included SSA, IFN‐alpha, chemotherapy and molecular targeted therapy. The costs for prescription drugs were based on data in the Swedish Prescribed Drug Register and included both patient co‐payment and the reimbursed cost.

The cost for transportation to and from a healthcare visit or admission related to metastatic GEP‐NET, carcinoid syndrome or carcinoid heart disease was estimated to be €31, derived from a previous Swedish publication (Bjorholt, Andersson, Kahan, & Ostergren, 2002).

The cost of lost productivity encompassed healthcare visits/admissions, sickness absence and mortality due to metastatic GEP‐NET, carcinoid syndrome or carcinoid heart disease. These costs were estimated only among patients aged <65 years (i.e., the standard Swedish retirement age). Production loss from absences due to healthcare use was estimated to be 2 hr per outpatient visit and the duration of admission for inpatient admissions. Only sickness absence periods lasting >14 days are reported in the Register on Sickness Absence (thus, shorter sickness absence periods were not captured). For these periods, the initial 14 days were included in the calculation of lost productivity. Production loss due to mortality was estimated from the deaths occurring in 2013, in which the cause of death was metastatic GEP‐NET, carcinoid syndrome or carcinoid heart disease. The costs were estimated until the point in time when the patient would have reached 65 years of age to balance the lack of data on production loss in 2013 due to deaths occurring prior to 2013. The costs occurring after 2013 were discounted at a rate of 3% per annum. The costs for lost productivity were measured with the human capital approach based on average national wages and social security contributions (Statistics Sweden, 2015). The annual cost of production loss was calculated to be €52,927 (year 2015).

All costs were adjusted to 2015 levels using the consumer price index and were presented in € (average exchange rate in 2015: €1 = 9.356 SEK). For brevity, €1K is used to indicate €1,000.

### 2.4 Statistical analyses

Standard descriptive statistics were used to summarise the data. For selected subgroup analyses, 95% confidence intervals (CIs) are presented. All analyses were performed in SAS® version 9.4 (Cary, NC, USA).

### 2.5 Ethical considerations

The study was approved by the Regional Ethical Review Board at the University of Gothenburg (Dnr 218‐15). For ethical reasons, the number of patients was presented as “<5” when the exact number of patients was 1–4 so that no individual could be identified.

## 3 RESULTS

### 3.1 Patient characteristics

The study population encompassed 478 patients who had been diagnosed with metastatic GEP‐NET (grade 1 or 2) in Sweden from 1 July 2005 to 31 December 2013, who were alive on 1 January 2013, and who had used healthcare resources for metastatic GEP‐NET, carcinoid syndrome or carcinoid heart disease in 2013.

The characteristics of the patients at diagnosis are shown in Table 1. The mean age at diagnosis was 64 (standard deviation [*SD*] = 11) years, and 51% (*n* = 243) were men. On 1 January 2013, 39% (*n* = 188) of the sample were aged <65 years. The most common primary tumour site was the small intestine (80%; *n* = 383), followed by the pancreas (10%; *n* = 49). Among the patients with known metastatic sites (39%; *n* = 186), the most common metastatic sites at diagnosis were the liver (57%; *n* = 106) and lymph nodes (46%; *n* = 86). Carcinoid syndrome was diagnosed in 41% of patients (*n* = 198) within 6 months of diagnosis.

**Table 1:** Characteristics of the study population (*n* = 478)

Age at diagnosis (years), mean (*SD*)	63.8 (11.2)
Sex, *n* (%)	
Male	243 (50.8)
Female	235 (49.2)
Tumour site, *n* (%)	
Small intestine	383 (80.1)
Pancreas	49 (10.3)
Other (stomach, colon, rectum)	46 (9.6)
Metastatic sites (most common), *n* (%)	
Liver	106 (57.0)
Lymph nodes	86 (46.2)
Intestine and peritoneum	20 (10.8)
Missing data, *n* a	292
Carcinoid syndrome^b^, *n* (%)	198 (41.4)

a Data on metastatic site were missing for a majority of patients (*n* = 292). Percentages were based on the number of non‐missing observations. A patient could have more than one metastatic site registered.

b ICD‐10 code E34.0 within 6 months from GEP‐NET diagnosis.

### 3.2 Cost‐of‐illness

The direct medical cost, direct non‐medical cost and cost of production loss due to metastatic GEP‐NET (including carcinoid syndrome and carcinoid heart disease) in Sweden in 2013 was €12,189K, which corresponds to an average cost of €25.5K per patient (Table 2). The direct medical cost constituted the largest proportion of the total costs (77%), followed by the cost of production loss (22%) and the direct non‐medical cost (<1%).

**Table 2:** Cost‐of‐illness of metastatic GEP‐NETs (grades 1 and 2) in Sweden per year

	Total cost (€1K)	Per cent of total cost
Direct medical costs	9,419	77
Healthcare resource use	4,326 (46% of direct medical costs)	35
Drugs	5,093 (54% of direct medical costs)	42
Direct non‐medical costs	57	<1
Cost of production loss	2,713	22
Healthcare visits/admissions	272 (10% of production loss costs)	2
Sickness absence	1,399 (52% of production loss costs)	11
Mortality	1,042 (38% of production loss costs)	9
Total	12,189	100

Note

€1K is used to indicate €1,000.

The total direct medical cost was estimated to be €9,419K, and the largest contributor was drugs related to GEP‐NETs (mainly SSA), constituting 54% of the total direct medical costs. The average cost per patient who had purchased each drug type was €15K for SSA (purchased by 65% of the patients), €4K for interferon alpha (10%), €3K for chemotherapy (3%) and €20K for molecular targeted therapy (3%). The remaining 46% of the direct medical costs were due to healthcare resource use. Overall, 13% of the patients had at least one surgery for metastatic GEP‐NET in 2013, at a total cost of €1,047K. The most common medical interventions were healthcare visits for administration of a pharmacological therapy for GEP‐NET (59 interventions; €2.4K per intervention), PRRT (50 interventions; €6.1K per intervention) and hepatic artery embolisation (35 interventions; €6.6K per intervention). The total cost of imaging related to metastatic GEP‐NETs was €470K, mainly from computed tomography (CT; 36% of the total cost of imaging; €2.4K per imaging), ultrasound (US; 22%; €3.6K per imaging) and scintigraphy (20%; €5.6K per imaging). Over 90% of patients had at least one specialist outpatient visit for metastatic GEP‐NET (with none of the listed interventions or procedures), at a total cost of €516K (Table 3).

**Table 3:** Direct medical costs of metastatic GEP‐NETs (grades 1 and 2) in Sweden per year

	Patients with at least one event, n (%)	Number of events	Cost (€1K)	Per cent of direct medical costs
Healthcare resource use	468 (98)		4,326	46
*Surgical interventions*	64 (13)	68	1,047	11
Surgery of primary tumour site	30 (6)	30	453	5
Surgery of metastatic sites	11 (2)	12	158	2
Concomitant surgery (primary and metastatic sites)	26 (5)	26	437	5
*Medical interventions*	65 (14)	166	830	9
Loco‐regional	31 (7)	47	368	4
HAE	21 (4)	35	230	2
RFA	10 (2)	12	138	1
PRRT	22 (5)	50	305	3
External radiotherapy	4 (1)	10	15	0
Administration of pharmacological treatment	13 (3)	59	142	2
*Diagnosis ascertainment*	10 (2)	10	125	1
Imaging	92 (19)	138	470	5
Scintigraphy	16 (3)	17	96	1
Magnetic resonance imaging (MRI)	6 (1)	6	7	0
Computed tomography (CT)	50 (11)	72	171	2
Ultrasound (US)	27 (6)	29	103	1
Other imaging	12 (3)	14	92	1
Examinations	37 (8)	45	243	3
Other outpatient visits	442 (93)	1 301	516	5
Other inpatient admissions	118 (25)	214	1,097	12
Drugs	326 (68)		5,093	54
SSA	311 (65)	2,493	4,630	49
IFN‐alpha	46 (10)	273	189	2
Chemotherapy	12 (3)	89	32	0
Molecular targeted therapy	12 (3)	63	241	3
Total			9,419	

Note

Events refer to healthcare visits, admissions or number of drug purchases.

€1K is used to indicate €1,000.

The total cost from production loss was estimated to be €2,713K among patients aged <65 years (*n* = 188 on 1 January 2013). Sickness absence constituted 52% of the total cost from production loss, followed by mortality (38%) and absence for specialist outpatient visits and inpatient admissions (10%; Table 4). There were a total of 9,647 days of sickness absence in 2013, corresponding to an average of 189 days per patient among those with at least one period of sickness absence (*n* = 51). Production loss from mortality was estimated to be €1,042K, based on five deaths in 2013.

**Table 4:** Costs of lost productivity for metastatic GEP‐NETs (grades 1 and 2) in Sweden per year

	Number of patients (% of patients <65 years; *N* = 188)	Costs (€1K)	Per cent of production loss costs
Absence due to healthcare use	180 (96)	272	10
Outpatient visits	172 (92)	235	9
Inpatient admissions	90 (48)	37	1
Sickness absence	51 (27)	1,399	52
Mortality	5 (3)	1,042	38
Total		2,713	

Note

€1K is used to indicate €1,000.

### 3.3 Subgroup analyses by tumour site

The total annual cost per patient was €24.8K (95% CI €21.6K–€28.1K) in small intestinal NET, €37.3K (95% CI €23.3K–€51.3K) in pancreatic NET and €18.6K (95% CI €12.6K–€24.5K) among those with GEP‐NETs at other sites. The total annual cost in small intestinal NET and pancreatic NET was significantly different (*p* = 0.018; Table 5). The cost of drugs per patient was highest for those with small intestinal NETs. SSA was purchased by 73% of patients with small intestinal NET (per‐patient cost: €11.0K), 31% of patients with pancreatic NET (€4.1K) and 37% of those with other GEP‐NETs (€4.9K). The cost of molecular targeted therapy per patient was €4.1K for those with pancreatic NETs (purchased by 20% of the patients), while the cost was €0.1K and €0.0K among patients with small intestinal NETs and other NETs, respectively.

**Table 5:** Costs of metastatic GEP‐NETs (grades 1 and 2) in subgroups by tumour site, in Sweden per year (cost per patient)

Cost per patient (€1K); 95% CI	Small intestine (*n* = 383)	Pancreas (*n* = 49)	Other (*n* = 46)
Direct medical costs	19.9 (18.1–21.7)	21.9 (16.1–27.7)	15.8 (10.5–21.0)
Healthcare resource use	8.4 (6.9–9.8)	13.0 (8.5–17.5)	10.5 (5.8–15.3)
Drugs	11.5 (10.5–12.5)	8.9 (5.0–12.8)	5.3 (3.0–7.5)
Direct non‐medical costs	0.1 (0.1–0.1)	0.2 (0.1–0.2)	0.1 (0.1–0.2)
Costs of production loss	4.8 (2.5–7.1)	15.2 (2.8–27.6)	2.7 (0.1–5.2)
Healthcare visits/admissions	0.5 (0.4–0.6)	1.0 (0.6–1.5)	0.7 (0.3–1.0)
Sickness absence	2.6 (1.7–3.6)	6.0 (1.5–10.5)	2.0 (0.0–4.5)
Mortality	1.7 (0.0–3.6)	8.1 (0.0–19.9)	0.0 (0.0–0.0)
Total	24.8^a^ (21.6–28.1)	37.3^a^ (23.3–51.3)	18.6 (12.6–24.5)

Notes

€1K is used to indicate €1,000.

a The total cost of small intestinal NET and pancreatic NET was significantly different (*p* = 0.018), as determined with a two‐tailed *t* test conducted at the 0.05 significance level.

The cost of production loss (included only for patients <65 years) per patient was highest in pancreatic NET, and this was observed for all categories of production loss. The percentage of patients aged <65 years on 1 January 2013 was 37% in small intestinal NET, 53% in pancreatic NET, and 44% in other NET.

## 4 DISCUSSION

The societal cost‐of‐illness of grades 1 and 2 metastatic GEP‐NETs was assessed among 478 patients. The annual economic burden was estimated to be €12,189K per year in Sweden, which corresponds to an annual per‐patient cost of €25.5K. The direct medical cost constituted the largest part of the total costs. Within the direct medical costs, drugs for the treatment of GEP‐NET were the main cost driver (mainly SSA). Production loss from sickness absence was the largest contributor to the indirect costs, followed by production loss due to mortality and absence due to healthcare use. The present study is most likely the largest societal cost‐of‐illness study ever performed on GEP‐NETs.

The annual per‐patient cost varied by tumour site and was highest among patients with pancreatic NET, followed by small intestinal NET, and lastly other GEP‐NET. The cost of production loss was highest in patients with pancreatic NET, and this finding was observed for all categories of production loss, although it was most pronounced for production loss due to mortality. While this result was based on only a few observations (5 deaths in total), it is consistent with previous findings showing that survival is poorer among patients with pancreatic NET than among patients with other GEP‐NET (Lawrence et al., 2011). Furthermore, a larger proportion of the patients with pancreatic NET were aged <65 years in 2013 (production loss was assessed only among patients aged <65 years). The cost of drugs was highest in small intestinal NET, which was mainly due to a more prevalent use of SSA among these patients. Patients with pancreatic NET had a higher cost of molecular targeted therapy than patients with small intestinal NET or other NET. This finding is also consistent with the prevailing guidelines from the European Neuroendocrine Tumour Society (ENETS; Pavel et al., 2016).

There are only a few previous studies of the costs associated with GEP‐NETs, and the majority of published studies are cost‐effectiveness analyses focusing on comparing specific procedures or therapies (reviewed in Chau, Casciano, Willet, Wang, & Yao, 2013). Comparisons of cost‐of‐illness estimates should be performed with caution, given the variations in the healthcare systems among countries, the perspective in which the costs are estimated (e.g., from a societal or healthcare perspective), the resources covered and the methods applied to assess resources and costs. To set the annual per‐patient cost of €25.5K from the perspective of other reported costs for gastrointestinal cancers, studies on the specific cost for pancreatic cancer report annual estimates of ~€49K and €78K from Germany and Sweden respectively (Carrato et al., 2015). A recent Finnish study on resource use for colorectal cancer reports a total cost of ~€40K per patient and year (Farkkila et al., 2015), although, as discussed above, differences in methods and scope in relation to the current study highlight the need for caution in making comparisons. For gastrointestinal cancers in general, in the absence of adequate annual per‐patient cost estimates, a report on costs for cancer in Sweden from 2016 indicates comparative total per‐patient costs based on incidence for cancers of the pancreas, liver, bile duct, stomach and oesophagus (Lundqvist, Andersson, & Steen Carlsson, 2016). Additionally, the per‐patient cost for breast and prostate cancer in Sweden seems to be comparable to the per‐patient cost reported in the present study, based on results presented in the 2016 report (Lundqvist et al., 2016). The total cost of cancer overall in Sweden has been estimated to be approximately €4,051 million (adjusted to 2015 levels; Jönsson & Wilking, 2007).

The main strength of the present study was that it was based on the use of real‐world data from national registers. The Nordic countries provide excellent opportunities for register‐based studies. All Swedish citizens are included in the registers, and the data are based on clinical practice. The linkage between registers is performed with high precision due to the unique personal identification numbers. Since the data are based on clinical practice, the diagnostic coding practices may vary among hospitals and/or physicians. The register data on healthcare use include specialised health care only; thus, resource use in primary care was not captured. Additionally, drugs used for complications from metastatic GEP‐NET, for example, carcinoid heart disease or diarrhoea, as well as non‐prescription drugs purchased over‐the‐counter, were not included. Drugs prescribed and purchased by the patient at a pharmacy were included, with only limited or no data available on drugs provided directly in connection with administration (e.g., chemotherapy). The total cost‐of‐illness estimate may also have been underestimated, since it is likely that not all patients in Sweden with grades 1 and 2 metastatic GEP‐NETs were captured in this study. The findings suggest the underreporting of data on metastatic status, with the consequence that some patients who had metastases at diagnosis may not have received such a diagnosis in the registers and would therefore not have been included in the study population. The exclusion of patients with probable grade 3 tumours may have also led to the exclusion of some patients with grade 1 or 2 tumours.

The data collection period of this study, 1 July 2005–31 December 2013, is also a potential limitation. Since 2013, multiple guidelines and recommendations have been published, and clinical practice for the treatment of GEP‐NETs has evolved (Niederle et al., 2016; Pavel et al., 2016).

The results presented in this study could form the basis of future decision making for healthcare resource allocation or in cost‐effectiveness analysis of emerging therapies for GEP‐NET.

## 5 CONCLUSIONS

The annual economic burden of grades 1 and 2 metastatic GEP‐NETs on society in Sweden was estimated to be €25.5K per patient and year. The direct medical costs constituted the largest part of the total cost, followed by costs from lost productivity.
